# Endocrine Dysfunction After Traumatic Brain Injury: An Ignored Clinical Syndrome?

**DOI:** 10.1007/s12028-022-01672-3

**Published:** 2023-02-14

**Authors:** Charu Mahajan, Hemanshu Prabhakar, Federico Bilotta

**Affiliations:** 1 Department of Neuroanaesthesiology and Critical Care, All India Institute of Medical Sciences, New Delhi, India; 2 Department of Anesthesiology, Policlinico UmbertoI Hospital, “Sapienza” University of Rome, Rome, Italy

**Keywords:** Traumatic brain injury, Pituitary dysfunction, Hypopituitarism, Hypothalamic-pituitary dysfunction, Adrenal insufficiency, Growth hormone deficiency

## Abstract

Traumatic brain injury (TBI) incurs substantial health and economic burden, as it is the leading reason for death and disability globally. Endocrine abnormalities are no longer considered a rare complication of TBI. The reported prevalence is variable across studies, depending on the time frame of injury, time and type of testing, and variability in hormonal values considered normal across different studies. The present review reports evidence on the endocrine dysfunction that can occur after TBI. Several aspects, including the pathophysiological mechanisms, clinical consequences/challenges (in the acute and chronic phases), screening and diagnostic workup, principles of therapeutic management, and insights on future directions/research agenda, are presented. The management of hypopituitarism following TBI involves hormonal replacement therapy. It is essential for health care providers to be aware of this complication because at times, symptoms may be subtle and may be mistaken to be caused by brain injury itself. There is a need for stronger evidence for establishing recommendations for optimum management so that they can be incorporated as standard of care in TBI management.

## Introduction

Traumatic brain injury (TBI) incurs substantial health and economic burden, as it is the leading reason for death and disability globally. Fall from height and motor vehicle crashes are the two most common causes of TBI, the latter generally affecting the economically productive population. Acute brain injury not only causes direct brain injury but also affects distant organs; the extent of extracerebral involvement depends on the severity of the injury. Endocrine abnormalities are no longer considered a rare complication of TBI, and the reported prevalence is variable across studies, depending on the time frame of injury, time and type of testing, and variability in hormonal values considered normal across different studies. The prevalence of hypopituitarism also changes over time. The pooled prevalence of pituitary dysfunction has been estimated to be around 27.5–32% [1, 2]. This is slightly less than the overall prevalence of pituitary dysfunction after aneurysmal subarachnoid hemorrhage, which is around 49% [3]. About one third of patients have persistent anterior pituitary disorder [4]. About 19.8–25.3% of patients with TBI have single pituitary axis involvement; involvement of multiple axes is less frequent (6.7–7.7%) [1, 2]. Overall, deficiency of anterior pituitary hormones is more significant than posterior pituitary hormone deficiency in both adults and children. The risk of hypothalamic–pituitary dysfunction after pediatric TBI has been noted to be higher in female patients and to peak at earlier ages compared with male patients, whereas in adult patients with TBI, sex does not seem to be related to adrenal insufficiency [5, 6]. After TBI in children, the incidence and prevalence of hormonal dysfunction peaked between the ages of 7 and 11 years, about 2 years after injury [5].

The symptoms may be mild and nonspecific, which may lead to a delay in the diagnosis. Moreover, it may remain unidentified in comatose patients or mechanically ventilated patients. Increased age, severity of TBI, cortical contusions, intracranial hemorrhage, seizures, and basal skull fractures are associated with a greater risk of developing anterior pituitary disorders [4]. However, not only severe TBI but also repeated sport-related head injuries are associated with pituitary dysfunction [7]. Serum sodium levels, urinary volume, and diffuse axonal injury are independent predictors of adrenocortical insufficiency after TBI [5]. Patients with TBI may experience endocrine dysfunction in both acute and chronic phases. First described in 1918 by Cyran et al., in patients with TBI, neuroendocrine dysfunction has been increasingly associated with not only physiological changes but also cognitive and behavioral alterations [8, 9]. Cognitive impairment associated with neuroendocrine dysfunction has also been recognized in patients with aneurysmal subarachnoid hemorrhage (aSAH) [10]. In the chronic state, cognitive changes, along with overlapping symptoms related to posttraumatic stress disorder, may further pose a diagnostic dilemma.

In the present review, we report evidence on the endocrine dysfunction that can occur after TBI. Several aspects, including pathophysiological mechanisms, clinical consequences/challenges, timing of screening and diagnosis, principles of therapeutic management, and insights on future directions/research agenda, will be presented.

## Pathophysiological Review

**Fig. 1:**
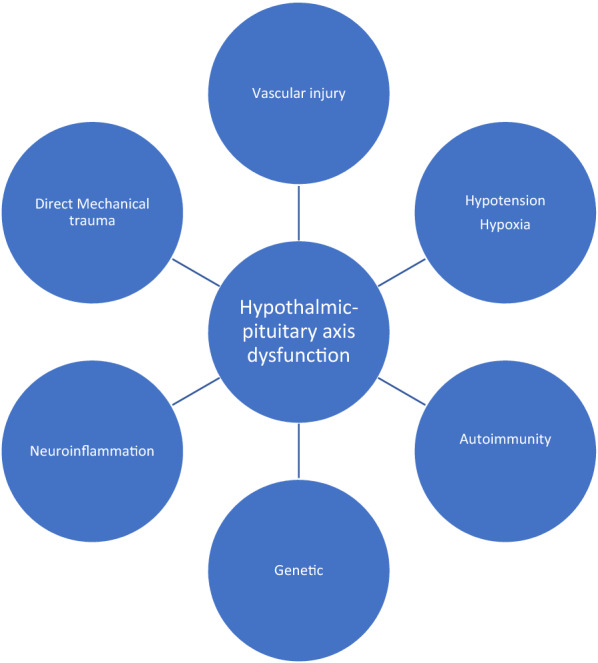
Main pathophysiological mechanisms of hypothalamic–pituitary axis dysfunction after traumatic brain injury

The hypothalamic–pituitary–adrenal (HPA) axis forms the backbone of the endocrine system and is responsible for systemic homeostasis. The main pathophysiological mechanisms of dysfunction are summarized in Fig. 1. The mechanical force of TBI can cause direct damage to the hypothalamus, pituitary stalk, or pituitary gland, which is situated at the base of the skull. The anterior pituitary is composed of pars distalis, pars tuberalis, and pars intermedia. Pars distalis forms most of the adenohypophysis involved in hormone secretion. The blood supply to the anterior pituitary is largely from the internal carotid arteries that give rise to superior and inferior hypophyseal arteries. The superior hypophyseal artery supplies the pituitary stalk and penetrates the median eminence of the hypothalamus to form a capillary plexus. The hypothalamic nuclei secrete releasing/inhibitory factors near the median eminence, from where they enter these capillaries that run down the pituitary stalk as portal vessels to reach the anterior pituitary. These act on the pituitary to release prolactin, adrenocorticotropic hormone (ACTH), thyroid-stimulating hormone (TSH), gonadotropins (follicle-stimulating hormone [FSH] and luteinizing hormone [LH]), and growth hormone (GH). The posterior pituitary comprises axonal projections from the hypothalamus, which stores and releases hormones (oxytocin and vasopressin) and receives its blood supply from the inferior hypophyseal artery. The long hypophyseal vessels that drain the capillary bed of the pituitary stalk traverse a longer path and pass through the diaphragma sellae, making it vulnerable to mechanical injury during head trauma or compressive effect due to raised intracranial pressure (ICP). This can cause ischemic necrosis of the adenohypophysis, resulting in hypopituitarism [11]. These vessels supply the anterolateral portion of the pituitary that contains somatotrophs and gonadotrophs, which explains the pattern of early hormonal loss [12]. Deficiency of GH is most commonly seen, followed by deficiency of ACTH, gonadotropins, and TSH. The short hypophyseal portal vessels drain the capillaries below the diaphragma sellae (near the lower infundibulum), supply the medial and anterior adenohypophysis, and run less chance of disruption and permanent damage. As a result, corticotropes and thyrotropes supplied by short portal vessels are less frequently involved. Low blood pressure, anemia, and hypoxia can cause ischemic changes of the gland. Injury to the stalk also affects hypothalamic neural regulation of the pituitary.

Central diabetes insipidus (DI) after TBI can occur because of direct injury to the hypothalamus or posterior pituitary gland. Edema of the hypothalamus or posterior gland can result in onset of transient DI, whereas injury to paraventricular and supraoptic hypothalamic nuclei, injury to the neurohypophysis, and transection of the pituitary stalk can cause permanent DI [13].

Raised ICP also predisposes to hypothalamic and pituitary apoptosis [14]. Genetic predisposition, neuroinflammation, and autoimmunity are also proposed mechanisms of pituitary dysfunction after TBI [15]. Presence of anti-pituitary antibodies and anti-hypothalamus antibodies in boxers experiencing chronic repetitive head trauma, points toward the role of autoimmunity [7]. Neuroinflammation induced by TBI alters blood brain barrier (BBB) permeability, and as a result, pituitary and hypothalamic antigens leak into circulation. Antibodies are thus induced in response, and anti-pituitary and anti-hypothalamus antibodies are formed. Higher values are associated with hypopituitarism, whereas negative titers correspond to recovery of pituitary function [15]. Genetic polymorphism of the *ApoE* gene has been found to affect outcome in TBI. The *ApoE3/E3* genotype is associated with reduced risk of hypopituitarism [16]. Most of the patients regain pituitary function over time, and this may take as long as several years. Genomic studies show that early detection of circulating messenger RNA can help identify the subset of patients with TBI who are vulnerable to development of hypopituitarism [17].

Administration of pharmacological drugs, such as pentobarbital, propofol, and etomidate, can also result in lower cortisol levels [18]. Moreover, acute illness can predispose to direct HPA dysfunction, altered cortisol secretion, and glucose metabolism.

Magnetic resonance imaging (MRI) after acute TBI reveals enlargement of pituitary gland dimensions in patients both with and without direct focal injury to the gland, pointing toward edematous changes in the pituitary gland [19]. Imaging studies have depicted reduced apparent diffusion coefficient in diffusion-weighted MRI of the pituitary gland depicting ischemia after TBI, and this was more significant in patients who developed hypopituitarism [20]. Pituitary volume reduction in imaging, possibly related to its necrotization, is another feature evident in the chronic phase that relates to hormonal loss [21].

## Clinical Challenges

The presentation can both be acute (within the first 2 weeks after TBI) and chronic (more than 3 months after TBI). The clinical picture may vary from mild, nonspecific features to life-threatening conditions. In the acute phase, any mental confusion, muscle weakness, or delirium due to pituitary hormonal imbalance might not be recognizable in patients with altered consciousness due to head trauma. Refractory hypotension in the absence of any other identifiable cause can be due to adrenal insufficiency. The gonadotropins and GH are most commonly deficient after TBI, but these might not be clinically evident immediately. During this period, hypocortisolism and DI requires appropriate treatment, with the rest of the hormonal deficiency not being diagnosed reliably in the early stage. The chronic phase often depicts GH deficiency (GHD) and gonadotrophin deficiency and less often depicts hypothyroidism and hypocortisolism. Anterior lobe hypopituitarism, which is more common after TBI, causes insulin resistance, hyperglycemia, increased abdominal fat deposition, higher waist circumference, and dyslipidemia [22]. Both GHD and thyroid deficiency can lead to a decrease in the basal metabolic rate. In addition to these, hypopituitarism after TBI affects neurocognition involving executive function, concentration, problem-solving ability, memory, and speech. These features can be caused by either head injury or GHD. Hypogonadism and hypothyroidism affect cognition and memory performance. Hypoadrenalism is associated with tiredness, inadequate response to stress, decreased memory, and mood disorders [23]. Emotional imbalance, depression, anxiety, and social issues are also seen. This affects the overall quality of life as well as delays rehabilitation of patients post TBI. We will be discussing the specific endocrine abnormalities in this section.

## Growth Hormone Deficiency

GH is a peptide hormone secreted from somatotropic cells located laterally in the anterior pituitary. These are dependent on blood supply from long portal vessels, predisposing them to injury and early loss of function after TBI (discussed earlier).

GHD has been found to be the most common pituitary hormone deficiency at 1, 3, and 5 years after TBI [24]. Tanriverdi et al. studied 52 patients with TBI and found prevalence to be 20.4% when assessed within 24 h and 37.75% in chronic stage (12 months after injury) [25]. However, prevalence of GHD varies across studies, depending on the severity of injury, age, time, and method of assessment. It varies from 2 to 30% in the acute stage [25–27] and persists in 10–63.6% of patients with TBI in the chronic stage [28]. It was observed that most pituitary hormone deficiencies improved over time, but at 5 years, still 28% of patients post TBI had GHD [24].

Compared with in adults, GHD in children ranges from 4 to 31% [29]. GHD and central precocious puberty may appear 3–4 years after severe TBI in children [30]. Precocious puberty results from loss of inhibitory action of extrahypothalamic areas on gonadotropin secretion. In the chronic phase, metabolic alterations and cognitive disorders are more appreciable. Young children may be particularly vulnerable to endocrine dysfunction because the brain is in the developmental stage during childhood. Unrecognized HPA axis dysfunction in children can result in delayed or absent puberty, short stature, poor muscle development, excess subcutaneous fat, adrenal insufficiency, and metabolic disorders. GHD after TBI has been seen to associate with poor quality of life, and in general, hypopituitarism after TBI is associated with poor outcome. Table 1 summarizes the clinical features of pituitary hormone deficiencies.

**Table 1:** Clinical features and diagnostic tests for hypopituitarism

Anterior pituitary hormones	Clinical features	Diagnostic tests
GH deficiency	Decreased exercise capacity, increased truncal obesity, reduced muscle mass and strength, osteoporosis, dyslipidemia, anxiety, depression, sleep impairment, psychosocial problems Children: dwarfism, poor muscle development, excess subcutaneous fat, delayed puberty	IGF-I level is less than normal for age- and sex-adjusted values Dynamic tests [31] Insulin tolerance test: intravenous human regular insulin 0.1–0.15 IU/kg results in peak GH at 120 min < 5.0 μg/l GHRH + Arg: ≤ 11.5 μg/l Glucagon stimulation test: glucagon 1–1.5 mg intramuscular results in peak GH at 180 min < 3 μg/l (when BMI < 30) and < 1 μg/l (when BMI > 30) Macimorelin stimulation test: Macimorelin 0.5 mg/kg in 1 ml/kg water orally results in GH level < 2.8 μg/l
Gonadotropin deficiency	Infertility, menstrual disturbances, osteoporosis, sexual dysfunction, osteoporosis, reduced muscle mass, loss of hair, galactorrhea, breast atrophy, cognitive disorders, fatigue	Males: low testosterone, low LH/FSH, and high prolactin levels Females: low estrogen, low FSH/LH, and high prolactin levels; progesterone challenge in secondary amenorrhea
ACTH deficiency	Generalized weakness, nausea, weight loss, anorexia, myalgia, dizziness, orthostatic hypotension, hypoglycemia, hyponatremia, eosinophilia, anemia, arthralgia	8 a.m. cortisol value < 3.5 μg/dl If value between 3 and 15 μg/dl, it requires stimulation tests 8 a.m. ACTH value is low Cosyntropin stimulation test: Low dose (1 μg IV bolus): cortisol at 30 min < 18 μg/dl High dose: 250 μg IV bolus: cortisol at 60 min < 18 μg/dl Insulin tolerance test (not preferred in acute TBI patients): cortisol at 45 min is < 18 μg/dl
TSH deficiency	Cold intolerance, fatigue, bradycardia, hypotension, weight gain, menstrual disorders, constipation, depression, hyperlipidemia, hyponatremia	Low values of TSH and free thyroxine
Post pituitary hormones		
ADH deficiency	Polyuria, polydipsia, nocturia	Polyuria > 3–4 l/day or > 50 ml/kg/day in older children or adults S.Na > 145 mEq/l Urine osmolality < plasma osmolality Urine osmolality < 300 mOsm/kg Urine-specific gravity < 1.005 Water deprivation test (not suitable for acute brain injured patients, can be done in chronic stage): urine osmolality < 700 mOsm/kg or ratio of urine to plasma osmolality < 2

*IGF 1* insulin growth factor 1, *GH* growth hormone, *GHRH* growth hormone releasing hormone, *LH* luteinizing hormone, *FSH* follicle stimulating hormone, *ACTH* adrenocorticotropin hormone, *TSH* thyroid stimulating hormone, *TBI* traumatic brain injury, *ADH* antidiuretic hormone, *BMI* body mass index

Testing for a low plasma insulin growth factor 1 (IGF-1) level is usually performed when diagnosing GHD. However, in acute trauma, the levels may not be reliable, and the test lacks sensitivity to screen patients with TBI. For diagnosing GHD, a low IGF-1 level warrants further dynamic testing unless it is associated with deficiency of three other pituitary hormones. GHD requires dynamic testing because GH secretion is of pulsatile and episodic nature. The insulin tolerance test (ITT) is generally considered a gold standard because it assesses both hypothalamic and pituitary function, but it runs the risk of causing neuroglycopenia and seizures after TBI, making it a less favored test to perform in these patients. GH-releasing hormone (GHRH) plus arginine and GHRH plus GH-releasing peptide 6 stimulate GH secretion and can be safely used as dynamic tests for GHD evaluation (Table 1).

## Gonadotropin Deficiency

Gonadotropins are peptide hormones, namely LH and FSH, secreted by the anterior lobe of the pituitary gland that regulate ovarian and testicular function. Dysfunction of the hypothalamic–pituitary–gonadal axis can result in low levels of sex steroids. In times of stress, as a compensatory mechanism of the body, the level of anabolic androgens decreases to conserve energy expenditure, enabling function of vital organs. Gonadotropin deficiency is the second most common hormonal deficiency, with a prevalence of about 40–80% in adults, but it is likely to be transient and much less prevalent in children [25, 27, 32]. In the chronic stage, the prevalence ranges from 2 to 32% [33], with 4% of the patients still having gonadotropin deficiency 5 years after TBI [24]. Agha et al. studied 50 adult patients with TBI 12 days (median) after injury and found 80% to have low serum gonadal sex steroid levels and low gonadotropin levels [27]. The authors also found a significant positive correlation of admission Glasgow Coma Scale (GCS) scores and functional outcome at 12 months with serum testosterone levels. By 12 months, 85% of these hypogonadal patients had recovered [27].

Hyperprolactinemia after TBI can be caused by stress (acute trauma), damage to the hypothalamus or pituitary stalk, or administration of antidopaminergic medications. Pituitary stalk compression can cause inhibition of negative feedback for prolactin secretion, causing hyperprolactinemia resulting in menstrual and sexual dysregulation. This causes hypogonadism by inhibiting secretion of LH and FSH, resulting in decreased levels of estrogen in women and testosterone in men. Hyperprolactinemia has been found to be present in 48% of hypogonadal patients in the acute phase, in 28% at 6 months, and in 33% at 12 months [34]. In children, it has been seen to be transient and of not much clinical significance [29]. The diagnosis of hypogonadism is tabulated in Table 1. Low levels of LH and FSH in the presence of low testosterone levels in men and low estrogen levels in women is diagnostic of hypogonadotropic hypogonadism. Along with these, serum prolactin levels should be measured in all patients.

## Secondary Adrenal Insufficiency

Patients with TBI develop secondary ACTH insufficiency because of the pituitary’s inability to release ACTH. Activation of HPA axis is an important protective mechanism to stress that triggers fight-or-flight response. Absence of this response can cause inflammation, hemodynamic instability, and poor outcome. Hypoadrenalism occurring early post TBI is a diagnostic a challenge because it can be a primary dysfunction because of relative adrenal insufficiency due to acute illness rather than pituitary dysfunction. Moreover, cortisol-binding globulin levels may change during acute illness, making total cortisol measurement a less reliable indicator of free cortisol. Cohan et al. conducted a prospective study in 80 patients with moderate–severe TBI to determine the prevalence and time course of adrenal insufficiency [18]. They defined adrenal insufficiency (AI) in patients with TBI as two consecutive cortisol levels < 15 μg/dl or a single cortisol level < 5 μg/dl. The first ACTH and cortisol levels were drawn within 24 h of injury, and subsequent samples were drawn at 6 a.m. and 4 p.m. to day 9 after injury. Transient relative AI occurred in 42 of 80 (53%) patients, and they were younger and sustained more severe injury and higher frequency of hypotension, hypoxia, and severe anemia than those without AI. Use of etomidate for intubation in patients with TBI was found to decrease adrenal response to the ACTH test for the first 24 h only and not beyond [18, 35]. Hyponatremia, hypoglycemia, hyponatremia, and a morning serum cortisol value less than 3.5 μg/dl strongly indicate adrenal insufficiency [36] (Table 1). In the presence of stress, value between 3 and 11 μg/dl may be considered as insufficient [37].

Adrenal insufficiency manifests as hypotension, hyponatremia, and hypoglycemia and is associated with significant morbidity and mortality. These patients usually require doses of vasopressors to maintain blood pressure, and a stress dose of steroids is found to be beneficial in them. Other clinical features are generalized weakness, nausea, weight loss, anorexia, myalgia, dizziness, anemia, arthralgia, and eosinophilia (Table1). Chronic AI is uncommon, and the HPA axis usually resumes normal function by 3–6 months. Chronic ACTH deficiency ranges from 8.2 to 9.9% in adults [2, 4] and is around 2% in children [24]. The decision to treat patients with a stress dose of glucocorticoids should be based on clinical features as well as on cortisol levels (Table 1). It is widely known that glucocorticoids in TBI are associated with poor outcome and should not be routinely administered. The administration of steroids should be based on the evaluation of cortisol level assessment and clinical profile of the patients. The insulin tolerance test can cause neuroglycopenia and even induce seizures in an already acutely injured brain and is not usually conducted in patients with TBI. ACTH stimulation tests with cosyntropin can be generally employed. An increase of less than 9 μg/dl in cortisol levels indicate likelihood of AI [38]. However, the ability of stimulation tests to diagnose adrenal insufficiency in the acute setting is questionable because they lack standardization [39]. Because diurnal variation of cortisol secretion is lost in acute illness [18], random cortisol levels of 10 μg/dl (when albumin level ≤ 2.5 g/dl) and 15 μg/dl (when albumin level > 2.5 g/dl) can be considered for initiation of glucocorticoid treatment [40].

**Fig. 2:**
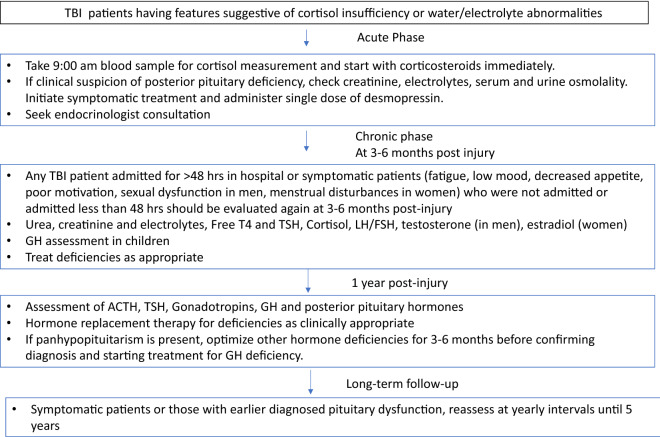
Clinical pragmatic suggestion for screening and diagnosis of hypopituitarism after traumatic brain injury (TBI). *TBI* traumatic brain injury, *GH* growth hormone, *TSH* thyroid stimulating hormone, *LH* leutinizing hormone, *FSH* follicle stimulating hormone, *ACTH* adrenocorticotropin hormone. (Adapted from Gilis-Januszewska et al. [44], Sundaram et al. [37], Glynn and Agha [56], Ghigo et al. [45], and Tanriverdi et al. [43], and Tan et al. [41].)

As a result of the challenges associated with diagnostic thresholds for AI, the British Neurotrauma group guidance does not recommend routine testing of pituitary hormones or cortisol measurement in the acute stage [41]. In the presence of clinical features of cortisol insufficiency, they recommend obtaining a random cortisol measurement followed by initiation of empirical replacement intravenous or intramuscular hydrocortisone 50 mg every 6–8 h or intravenous infusion. This is contrary to suggested practice by Glynn et al., who support routine screening for cortisol insufficiency in all patients with TBI, keeping in mind the morbidity associated with AI in these patients [42]. Tanriverdi et al. also recommends testing all patients with complicated mild, moderate, and severe TBI for ACTH deficiency [43]. On basis of the current literature and reliability of screening tests, we support testing patients with TBI for AI, only if clinical features are suggestive (Fig. 2).

## Diabetes Insipidus and Syndrome of Inappropriate Antidiuretic Hormone Secretion After Traumatic Brain Injury

Injury to the hypothalamus, posterior pituitary, or pituitary stalk may cause water and electrolyte abnormalities. DI occurs because of underproduction of antidiuretic hormone (ADH), leading to fluid and electrolyte imbalance. For diagnosing DI, exclusion of the effect of osmotic agents and hyperglycemia is paramount. The diagnostic criteria for DI are given in Table 1. The incidence of severe DI with gross hypernatremia was found to be 2.9% [46]. In the early period after injury, the overall incidence of DI is about 22–26% [27]. Patients with TBI have impaired consciousness, as well as nausea, vomiting, and pain, and receive osmotic diuretics for raised ICP, leading to decreased total intake. Occurrence of polyuria leads to much more water loss, resulting in a negative water balance. The consequent hypernatremia and dehydration add to the morbidity. It has been suggested that patients with features of acute DI should undergo screening for anterior pituitary dysfunction [45]. Though most of the times it is transient, still in around 6.9% of the patients with TBI, DI may persist even 6–36 months after injury [27].

Hyponatremia (Na level < 135 mmol/l) is the most common (20%) electrolyte abnormality seen after TBI [47]. Syndrome of inappropriate antidiuretic hormone secretion (SIADH) has been observed in 12.7% of patients with TBI in the acute phase [27]. It is also usually transient but can cause hyponatremia, increase cerebral edema, and even precipitate seizures. Acute ACTH deficiency can coexist in patients with TBI and is one of the reasons for hyponatremia. For making a diagnosis of SIADH, glucocorticoid deficiency, severe hypothyroidism, and the antiseizure drug carbamazepine need to be excluded. Patients who develop hypotension, hypoglycemia, and hyponatremia are likely to have ACTH deficiency and should be screened for it.

## Thyroid Stimulating Hormone Deficiency

Hypothyroidism is infrequent in the early stage and is difficult to recognize because of associated nonspecific features. Low T3 and T4 levels with normal TSH levels in the early stage can be due to injury itself and can persist for 2–3 weeks. This low-T3-syndrome-like state cannot reliably identify secondary hypothyroidism due to pituitary dysfunction. TSH deficiency is less frequent, being 4.1–6.2% 12 months after injury [2, 4]. In a long-term follow-up study of children with severe TBI, about 13% of patients had pituitary dysfunction (10% GHD, 3% TSH deficiency, and 2% ACTH deficiency) when followed for 1–7 years post TBI [30]. Experimental studies show thyroid hormones have a role in reducing brain edema and stimulating neural regeneration [48].

## Who and When to Screen

The absence of lesions in imaging modalities such as head computed tomography (CT) and head MRI does not rule out pituitary dysfunction. The cost of screening each and every patient with TBI for hormonal dysfunction is high and is not feasible. Also, during the acute phase, stress response and drugs such as etomidate, propofol, and pentobarbital can alter the hormonal levels. So we need to understand which patients with TBI should be screened for hormonal profile. Thus, patients with mild head injury are not routinely evaluated for pituitary function in the acute stage, unless they have symptoms suggestive of hypopituitarism. Because there is no concrete evidence that GH and gonadotropin replacement benefits in the early period after TBI, patients need not be routinely screened for these hormones in the early period. However, any patient with signs of adrenal insufficiency (refractory hypotension, hypoglycemia, hyponatremia) or electrolyte abnormality should be screened for hormonal deficiency [41].

The pooled prevalence of chronic hypopituitarism is higher in severe TBI compared with mild and moderate head injury [4]. Presence of diffuse axonal injury (DAI), skull fracture, basal skull fracture, diffuse brain swelling, evacuated hematoma, and multiple contusions are possible radiological risk factors for long-term pituitary dysfunction [4] and possibly need screening. Patients with moderate-to-severe TBI and clinical signs or symptoms associated with hypopituitarism should be screened for pituitary dysfunction [27]. Patients with complicated mild head injury (i.e., if they require hospitalization for at least 24 h, require intensive care unit admission, are of older age, had an abnormal head CT scan result [brain swelling, DAI, basal skull fracture, epidural/subdural hematomas, cranial vault fracture] on admission, have hypoxia/hypotension, have repetitive injuries [sports related], had polyuria/polydipsia during the acute phase, or have signs of pituitary dysfunction) thereafter should be evaluated for hormonal deficiency [43]. If central DI is suspected, serum creatinine, electrolytes, plasma glucose, and serum and urine osmolality tests should be checked before administering desmopressin [41]. Patients with suspected SIADH should be evaluated for renal, adrenal, and thyroid dysfunction. Any patient with TBI who presents with symptoms related to hypopituitarism should be screened for pituitary dysfunction. Patients not admitted or admitted for less than 48 h who have features consistent with pituitary dysfunction may also require screening [41]. Tanriverdi et al. suggest that patients with mild TBI who are discharged from emergency units and/or who have no loss of consciousness and/or posttraumatic amnesia of less than 30 min and patients with TBI in chronic vegetative states with low life expectancy need not be screened further [43].

Glynn and Agha suggest measurement of morning cortisol values for the first 7 days after TBI [42]. Tanriverdi et al. recommend measurement of cortisol in every patient on days 1–4 and on days 5–10 post injury in case of clinical suspicion. The authors recommend retesting at discharge (at least 2 weeks post TBI), at 6 months, and again at 12 months. If hormonal deficiency persists at 1 year, yearly reassessment until 5 years is suggested [43]. The time frame of assessment varies as per different authors, and thus we suggest a screening schedule based on the widely accepted opinion. Figure 2 highlights the screening schedule for diagnosis of hypopituitarism after TBI based on the current literature.

For chronic GHD and ACTH deficiency, stimulation tests are required for identifying somatotroph and corticotroph dysfunction (Table 1). Thyrotropin deficiency and gonadotropin deficiency are diagnosed by low values of the respective hormones. Similarly, diagnostic criteria for central DI are tabulated in Table 1. In the chronic phase, patients with moderate–severe TBI and symptomatic patients with mild TBI should be screened for HPA dysfunction [45, 49]. The best time of screening for hormonal deficiency is controversial, but screening between 3 and 6 months can help in early diagnosis and treatment [41]. Patients with abnormal test results at 3–6 months should be referred to endocrinology for detailed hormonal assessment along with dynamic tests [41]. Consensus is that clinical evaluation for hypopituitarism should be conducted routinely 3–6 months and 12 months following TBI [41, 50]. Usually by 12 months, transient alterations get corrected or some new deficiency might be identified, making it a good testing time point. In children with GHD, growth charts and metabolic profile needs to be followed up for a long time. This makes regular monitoring necessary every 6 months during the first year after TBI in children, followed by yearly follow-up [5]. Tan et al. recommend screening for pituitary dysfunction after 1 year only if patients are symptomatic; otherwise, no further action is required [41]. On the other hand, Tanriverdi et al. suggest reassessment at yearly intervals up to 5 years in patients with complicated mild TBI [43].

## Evidence for Management

In the acute stage of TBI, adrenal insufficiency and ADH deficiency need to be managed. The management of hypopituitarism following TBI involves hormonal replacement therapy. GHD, TSH deficiency, and gonadotropin deficiency are usually transient and do not require therapy in the acute stage. However, ACTH deficiency and ADH deficiency require prompt, appropriate management. Adrenal insufficiency requires administration of glucocorticoids for symptomatic improvement. ADH deficiency requires correction of the fluid and electrolyte imbalance, and if ADH deficiency is persistent, treatment with desmopressin is indicated. At 3–6 months, patients are assessed for ACTH, posterior pituitary hormone, TSH, and LH/FSH, and if hormone levels are deficient, appropriate replacement is required [42]. GHD and LH/FSH deficiency recover in many patients, making it imperative to test for these at 1 year post TBI. GH assessment may be postponed until 1 year after the injury, except in children who require earlier assessment [44]. GH replacement after TBI has been shown to improve cognition, especially memory and attention, and quality of life [51, 52], but the evidence for other parameters is not strong enough for mass generalization. In a recent systematic review, Szarka et al., identified 12 studies (264 patients with mild–moderate–severe TBI) of patients with or without GHD who received GH therapy [53]. Authors found that regardless of the GCS, GH therapy started in the chronic phase of TBI resulted in a moderate improvement in processing speed and memory, reduced the severity of depression, and markedly improved the quality of life. However, the small number of patients in the studies and variable neuropsychological tests used in studies indicate a need to conduct further multicentric controlled trials [54]. The data concerning the effect of GH administration on metabolic/cardiovascular risk factors and bone health show beneficial effect, yet the evidence is not strong enough to prove reduction of cardiovascular events or improvement in skeletal outcomes [50]. GH helps to improve rehabilitation of patients with TBI by improving socialization, decreasing depression, improving self-confidence [54]. The hormone replacement therapy is continued for 3–6 months as clinically appropriate, and periodic reassessments are done thereafter. It is necessary to test again at 1 year because patients may recover from deficiencies or new deficiencies may appear. Replacement sex steroid therapy is initiated after diagnosis is confirmed at 3–6 months and is continued in the first year after TBI. Hyperprolactinemia needs to be corrected first before starting sex steroid therapy. At 1 year, patients are retested, and, accordingly, a decision to continue or stop treatment is made. This therapy is essential for recovery and rehabilitation of patients with TBI.

## Future Directions/Research Agenda

While studying the possible biological signatures for TBI, there is also an unmet need to identify biomarkers for recognizing at-risk patients for pituitary dysfunction. A lower plasminogen activator inhibitor type 1 level at the time of injury was seen to be a predictor of late pituitary dysfunction [55]. On imaging, decreased apparent diffusion coefficient (ADC) in the pituitary correlates with hypopituitarism after injury [17]. However, more substantial evidence is required for identification of a reliable biomarker. Polymorphism of the *ApoE* gene also affects development of hypopituitarism [15]. Genome-wide association studies may help identify genetic variants susceptible to development of hypopituitarism after TBI as well as understand the responsiveness to hormonal replacement therapy, especially GH. Genotype–phenotype association studies can help better understand the pathophysiology and the treatment strategies for hypopituitarism after TBI. There is still a need for conducting randomized clinical trials to assess the beneficial effects of GH replacement therapy on recovery and outcome in GH-deficient patients post TBI. The large databases Collaborative European NeuroTrauma Effectiveness Research in Traumatic Brain Injury (CENTER-TBI) and International Mission for Prognosis and Analysis of Clinical Trials (IMPACT) can provide us with important data regarding hypopituitarism after TBI.

## Conclusions

The occurrence of pituitary dysfunction after TBI is no longer an ignored clinical entity and is increasingly being identified as the potential factor affecting long-term outcome and rehabilitation of patients after TBI. It is essential for health care providers to be aware of this complication because at times, symptoms may be subtle and may be mistaken to be caused by brain injury itself. There is a need for stronger evidence for establishing recommendations for optimum management so that they can be incorporated as standard of care in TBI management.
